# Pasteurized form of a potential probiotic *lactobacillus brevis* IBRC-M10790 exerts anti-inflammatory effects on inflammatory bowel disease in vitro

**DOI:** 10.1186/s12906-024-04576-1

**Published:** 2024-07-10

**Authors:** Ardeshir Ebrahiminejad, Abbas Akhavan Sepahi, Abbas Yadegar, Anna Meyfour

**Affiliations:** 1 Department of Microbiology, Faculty of Biological Sciences, Islamic Azad University, North Tehran Branch, Tehran, Iran; 2 Foodborne and Waterborne Diseases Research Center, Research Institute for Gastroenterology and Liver Diseases, Shahid Beheshti University of Medical Sciences, Tehran, Iran; 3 Basic and Molecular Epidemiology of Gastrointestinal Disorders Research Center, Research Institute for Gastroenterology and Liver Diseases, Shahid Beheshti University of Medical Sciences, Tehran, Iran

**Keywords:** *Lactobacillus brevis*, Inflammatory bowel disease, Proinflammatory cytokines, Pasteurized probiotics, Co-culture model, TNF-α

## Abstract

**Background:**

Inflammatory bowel disease (IBD) is a chronic, relapsing inflammatory disorder of the gastrointestinal system. So far, no treatment has been identified that can completely cure IBD. *Lactobacillus brevis* is hypothesized to be beneficial in preventing inflammation. This study aimed to evaluate the potential probiotic effects of live and pasteurized *L. brevis* IBRC-M10790 on the in vitro cell co-culture model of IBD.

**Methods:**

An in vitro intestinal model was established using a transwell co-culture system of Caco-2 intestinal epithelial cells and RAW264.7 macrophages. Inflammatory conditions were induced in RAW264.7 cells using lipopolysaccharide. The effects of live and pasteurized *L. brevis* IBRC-M10790 on inflammatory mediators and epithelial barrier markers were investigated.

**Results:**

*L. brevis* IBRC-M10790 was able to significantly decrease the proinflammatory cytokines (IL-6, IL-1β, and TNF-α) and increase the anti-inflammatory cytokine (IL-10) in the in vitro co-culture system. In addition, *L. brevis* increased adherens and tight junction (TJ) markers (ZO-1, E-cadherin, and Occludin) in Caco-2 intestinal epithelial cells. Based on the results, pasteurized *L. brevis* showed a higher protective effect than live *L. brevis.*

**Conclusions:**

Our findings suggest that live and pasteurized forms of *L. brevis* possess probiotic properties and can mitigate inflammatory conditions in IBD.

**Supplementary Information:**

The online version contains supplementary material available at 10.1186/s12906-024-04576-1.

## Introduction

Inflammatory bowel disease (IBD) is a group of chronic immune-mediated diseases in the gastrointestinal tract [1], which affects millions of people worldwide [2]. A set of factors, including environmental, immunological, genetic, and microbial agents have an important effect on the initiation and development of IBD [3]. Meta-analysis research has indicated reduced gut microbiota diversity in IBD patients, which is mainly due to a decrease in a variety of Firmicutes such as, *Clostridium leptum* [4], *Faecalibacterium prausnitzii* [5, 6], and *Lactobacillus* as well as a reduction in *Bifidobacterium* [7].

After the initiation of IBD, the intestinal epithelial barrier is harmed and macrophages begin to secrete proinflammatory cytokines such as TNF-𝛼, IL-1β, and IL-6 [8, 9]. One of the most important inflammatory stimuli that affect development of IBD is endotoxin lipopolysaccharide (LPS), which is present on the membrane surface of Gram-negative bacteria [10]. LPS stimulates macrophages, which increases the secretion of pro-inflammatory cytokines and inflammatory mediators [11]. Due to the importance of the role of probiotics on the host immune system, they have been widely studied and several studies have recently revealed fundamental concepts about the antioxidant and anti-inflammatory properties of probiotics [12–15]. Lactic acid bacteria (LAB) affect the host immunity [16]. It has been demonstrated that the gene expression of anti-inflammatory or pro-inflammatory cytokines such as IL-10, IL-12, and IL-6 could be induced by lactobacilli [17]. Heat-killed *Lactobacillus casei* and *Lactobacillus. fermentum* could enhance immune functions by increasing TNF-α levels in macrophages [18, 19]. *Lactobacillus* is one of the indicators of a healthy intestine, and it has previously been used to treat diseases related to digestive disorders, such as antibiotic-associated dysbiosis, IBD, and lactose intolerance [20–22]. Studies have shown that treatment with *Lactobacillus* species reduces immune responses and suppresses the overexpression of inflammatory factors in the host [23]. It has also been reported that heat-killed *L. casei* IMAU60214 could function as a probiotic and increase the phagocytic activity of macrophages [17].

Previously, studies have shown that the use of some live or heat-killed strains of *L. brevis* in the colitis-induced mice regulates proinflammatory factors and reduces the secretion of inflammatory mediators, leading to a reduction in the inflammation of the intestine [24]. Therefore, researchers hope that administration of probiotic strains of *L. brevis* in IBD patients can manage the inflammation to a great extent and prevent the disease complications [25]. In vitro IBD models could provide a robust platform to investigate the regulatory mechanisms of probiotics on colitis [26, 27].

In this study, a model of intestinal inflammation was created in vitro, in which a co-culture system of Caco-2 and RAW264.7 cells was used to examine the inhibitory effects of live and pasteurized forms of *L. brevis* IBRC-M10790 on the expression of proinflammatory cytokines.

## Materials and methods

### Bacterial strain and culture

*L. brevis* (IBRC-M10790) was gained from the Iranian Biological Resource Center (IBRC, Tehran, Iran). *L. brevis* (IBRC-M10790) was cultured anaerobically in the MRS agar. This strain was identified as LAB by Gram staining and catalase test using MRS (de Man, Rogosa and Sharpe) agar, and its biochemical and microbiological characteristics were further investigated to confirm the strain by different tests, including DNase, hemolysis, gelatinase, arginine dihydrolase, oxidase, and sulphide indole motility (SIM) assays. In addition, *L. brevis* IBRC-M10790 was confirmed by 16 S rRNA by PCR. Since some probiotic strains may produce enzymes that able them to inactivate antibiotics, it must be ensured that the strain does not potentially contribute to the development of antibiotic resistance [28, 29]; to confirm that, the disc diffusion method and Vitek-2 automatic system (bioMérieux) were used. Furthermore, acid pH tolerance and bile salts tolerance assays are necessary to certify that a probiotic species is able to survive and grow in the harsh human gut environment [30]. For the acid tolerance assay, the viability of *L. brevis* grown in MRS acid broth with different pH values of 1, 2, 3, 5.7, 6, and 8 was determined after 4 h of incubation at 37 °C. For the bile salts tolerance assay, *L. brevis* was incubated in bile salts medium with concentrations of 0.3% and 1% for 6 h at 37 °C, and then cultured on MRS agar plates. The growth rate of bacteria was recorded after 48 h.

### Pasteurization of *L. Brevis*

*L. brevis* was cultured in the MRS agar under anaerobic conditions. It was then inoculated into the new MRS broth with mild shaking until it reached an optical density of 1 at 600 nm. The bacterial mass was centrifuged and the sediments were washed twice with phosphate buffered saline (PBS). To prepare the pasteurized form, the bacterial suspension was heated at 70 °C for 30 min [31]. Cell suspensions obtained from live and pasteurized forms of *L. brevis* bacteria were morphologically examined by scanning electron microscope (SEM) and transmission electron microscope (TEM) [32].

### Cell lines and culture conditions

The RAW264.7 (murine macrophage cells) and Caco-2 (human colorectal adenocarcinoma) cell lines were purchased from IBRC and were cultured in DMEM. Fetal bovine serum (FBS) (10% v/v), streptomycin (50 µg/ml), penicillin (50 U/ml), and L-glutamine (2 mM) were added to both media, and then the cells were incubated at 37 °C in a humidified 5% CO_2_ incubator.

### Cytotoxicity evaluation of *L. Brevis*

To investigate the cytotoxicity of *L. brevis* IBRC-M10790, Caco-2 cells were cultured in 96-well microplates at 37 °C in 5% CO_2_. The concentration of cells in each well was 1 × 10^5^ cells. Live and pasteurized *L. brevis* IBRC-M10790 were then added to plates containing Caco-2 cells. Bacteria were added at different multiplicities of infection (MOIs) including 1, 10, 50, and 100. After 24 h of incubation, the 3-(4,5-dimethylthiazol-2-yl)-5-(3-carboxymethoxyphenyl)-2-(4-sulfophenyl)-2 H-tetrazolium (MTS) assay was performed according to the manufacturer’s protocol (Promega).The optical density (OD) was determined at an absorbance of 490 nm [33] using a multi-well plate reader (ELx800; BioTek), and cell viability was defined as follows [34]:

%Viable cells: [(treated cells OD) - (blank OD)] / [(control OD) - (blank OD)] x 100.

### Transwell co-culture system

An intestinal simulated model was created using a co-culture system of Caco-2 and RAW264.7 cells in vitro. To simulate IBD, Caco-2 cells were transferred (1.5 × 10^5^ cells per well) on the apical side of a 6-transwell insert plate (0.4 μm pore size; Corning CoStar Corp, USA) and differentiated for 21 days. The culture media were changed every three days. RAW264.7 cells were then transferred (1.5 × 10^5^ cells per well) on the basolateral side of 6-transwell plates [35, 36]. To evaluate the effects of live and pasteurized *L. brevis* IBRC-M10790 on the LPS-induced co-culture system, four experimental groups with different combinations of treatments were considered (Table 1). LPS with a concentration of 1 µg/ml was added to RAW264.7 cells to induce inflammation. Live or pasteurized *L. brevis* IBRC-M10790 was added to the apical side at a MOI of 10 and incubated for 24 h. The supernatants were collected from both sides to determine the amount of TNFα, IL-10, and IL-6. Finally, the cultured cells were collected for total RNA isolation and quantitative RT‑PCR (RT-qPCR) analysis.

**Table 1:** Treatment of LPS and *L. Brevis* bacteria in the co-culture system in different combinations

Co-culture	1	2	3	4
Apical side	Caco-2	Caco-2	Caco-2 + L.L *	Caco-2 + P.L * *
Basolateral side	RAW * * *	RAW + LPS	RAW + LPS	RAW + LPS

*L.L, live *L. brevis*

*** *P.L, pasteurized *L. brevis*

* * *RAW, RAW264.7

### Cytokine measurement

The levels of TNF-α, IL-6, and IL-10 in cell culture supernatants were determined using ELISA cytokine detection kits (Carmania Pars Gene Company, Kerman, Iran) according to the manufacturer’s instructions. The absorbance was read at 450 nm by a multi-well plate reader (BioTek, ELx800).

### RT‑qPCR

The RT-qPCR method using SYBR Green chemistry was used to detect mRNA expression levels of TNFα, IL-6, IL-1β, Occludin, E-cadherin, and ZO-1 in Caco-2 cells as well as TNFα and IL-6 in RAW264.7 cells. In summary, total RNA was extracted from cells using Trizol and chloroform reagents, and cDNA was synthesized by reverse transcription on the extracted mRNA using the cDNA synthesis kit (Favorgen^®^, Taiwan). PCR amplifications were performed using specific primers (Table 2). Quantitative RT-qPCR was performed according to the following steps: 95 °C (15 min), 40 cycles of 95 °C (20 s), 56 °C (30 s), and 72 °C (20 s). Relative mRNA expression levels were estimated by the comparative 2^−ΔΔCt^, and all gene expression data were normalized as compared to *β-actin* housekeeping gene [37].

**Table 2:** Primer sequences in RT-qPCR

Target cell	Target gene	Primer sequences (5´-3´)	Organism
Caco-2	IL-10	F: GGGTTGCCAAGCCTTATCG R: TCTCACCCAGGGAATTCAAATG	*Homo sapiens*
	IL-6	F: GCACTGGCAGAAAACAACCT R: TCAAACTCCAAAAGACCAGTGA	
	IL-1β	F: GCACGATGCACCTGTACGAT R: CACCAAGCTTTTTTGCTGTGAGT	
	TNFα	F: CCCAGGGACCTCTCTCTAATC R: ATGGGCTACAGGCTTGTCACT	
	Occludin	F: CCCATCTGACTATGTGGAAAGA R: AAACCGCTTGTCATTCACTTTG	
	E-cadherin	F: TGCTCTTGCTGTTTCTTCGG R: CTTCTCCGCCTCCTTCTTC	
	ZO-1	F: GGGAACAACATACAGTGACGC R: CCCCACTCTGAAAATGAGGA	
RAW264.7	IL-10	F: AATAACTGCACCCACTTCCC R: ACCCAAGTAACCCTTAAAGTCC	*Mus musculus*
	IL-6	F: CTCTGCAAGAGACTTCCATCC R: TTCTGCAAGTGCATCATCGT	
	IL-1β	F: CTTTGAAGAAGAGCCCATCCT R: GTCGTTGCTTGGTTCTCCT	
	TNFα	F: TCTGTCTACTGAACTTCGGG R: TTGGTGGTTTGCTACGAC	

### Western blotting analysis

For western blotting, harvested cells were lysed with RIPA buffer. The lysates were separated by centrifuging at 14,000 x g for 20 min at 4 °C. The Bradford Protein Quantification kit (DB0017, DNAbioTech, Iran) was used to determine protein concentration following the manufacturer’s instructions. An equal volume of 2X Laemmli sample buffer was mixed with the tissue lysates. After boiling for 5 min, lysates (20 µg) underwent SDS-PAGE and were subsequently transferred to a 0.2 μm immune-Blot™ PVDF membrane (Bio-Rad Laboratories, CA, USA). The membranes were then blocked with 5% BSA (Sigma Aldrich, MO, USA) in 0.1% Tween 20 for 1 h. Following that, the membranes were subjected to incubation with Anti-ZO-1 antibody (1/1000, Cat No: 5406 S, Cellsignal), E-cadherin antibody (1/1000, Cat No: ab308347, Abcam), and anti-β actin-loading control antibody (1/2500, Cat No: ab8227, Abcam) for 1 h at room temperature. Next, the membranes were washed three times with TBST and then incubated with goat anti-rabbit IgG H& L (HRP) (1/10,000, Cat No: ab6721). Subsequently, the membranes were exposed to enhanced chemiluminescence (ECL) for a period of 1–2 min. β-actin was used as a reference to normalize protein expression. Densitometry of protein bands were performed using the gel analyzer Version 2010a software (NIH, USA). The percentage area of each band was divided by the percentage area of its corresponding actin band. The resulting values were compared between groups, following the methodology described in our previous publication [38].

### Statistical analysis

Statistical analyses of data were conducted using SPSS 20 (SPSS, Inc., Chicago, IL) and Graph Pad Prism 5.0 (San Diego, USA). All the results obtained in this study were expressed as the mean ± standard error of the mean (SEM). Also, the data were analyzed using one-way ANOVA. *P-value* of < 0.05 was regarded as statistically significant.

## Results

### Characteristics of *L. Brevis* IBRC-M10790

The biochemical and microbiological characteristics of *L. brevis* IBRC-M10790 are reported in Table 3. This bacterium was negative in terms of DNase, catalase, gelatinase, arginine dihydrolase, oxidase, hemolysis, and SIM tests. According to the results obtained from the investigation of biochemical and microbiological characteristics, it was shown that *L. brevis* IBRC-M10790 potentially has the characteristics of a probiotic strain (Table 3). Furthermore, the results showed that among the 26 tested antibiotics, *L. brevis* IBRC-M10790 was resistant to oxacillin, vancomycin, cephalothin, cefazolin, and cefoxitin. The antibiotic resistance profile of probiotic strain is shown in Table 4. In addition, *L. brevis* IBRC-M10790 showed tolerance to various pH values (pH 2 and higher), and bile salt concentrations (1% and 0.3%) (Table 5).

**Table 3:** Biochemical and microbiological characteristics of *L. Brevis* IBRC-M10790

Characteristics	L. brevis
Cell shape	Bacillus form
DNase test	-
Catalase test	-
Hemolysis test	-
Gram staining	+
Gelatinase test	-
Arginine dihydrolase test	+
Oxidase test	-
SIM test*	-

*Sulfur, Indole, Motility (SIM)

**Table 4:** The results of antibiotics susceptibility testing of *L. Brevis* IBRC-M10790

Number	Antibiotics	Concentration	Resistance	Number	Antibiotics	Concentration	Resistance
1	Amikacin	30 µg	S	14	Ertapenem	10 µg	S
2	Amoxicillin	20 µg	S	15	Erythromycin	15 µg	S
3	Ampicillin	10 µg	S	16	Gentamicin	10 µg	S
4	Cefazolin	30 µg	R	17	Imipenem	10 µg	S
5	Cefepim	30 µg	S	18	Levofloxacin	5 µg	S
6	Cefotaxime	30 µg	S	19	Linezolid	30 µg	S
7	Cefoxitin	30 µg	R	20	Oxacillin	1 µg	R
8	Ceftriazone	30 µg	S	21	Piperacillin	100 µg	S
9	Cephalothin	30 µg	R	22	Tetracycline	30 µg	S
10	Chloramphenicol	30 µg	S	23	Tigecycline	15 µg	S
11	Ciprofloxacin	5 µg	S	24	Trimethoprim	5 µg	S
12	Clindamycin	2 µg	S	25	Vancomycin	30 µg	R
13	Doxycycline	30 µg	S				

**Table 5:** The results of bacterial growth (based on the number of colonies) after tolerating acidic and bile salt conditions

Bile and acid tolerance	pH 1	pH 2	pH 3	pH 5.7	pH 6	pH 8	Bile 0.3%	Bile 1%
Growth rate	-	+	++	+++	+++	+++	+++	+++

- = No colony, + = 0-100 colonies, ++ =300–500 colonies, +++ = >1000 colonies

### Electron microscopy observation of live and pasteurized *L. Brevis* IBRC-M10790

**Fig. 1:**
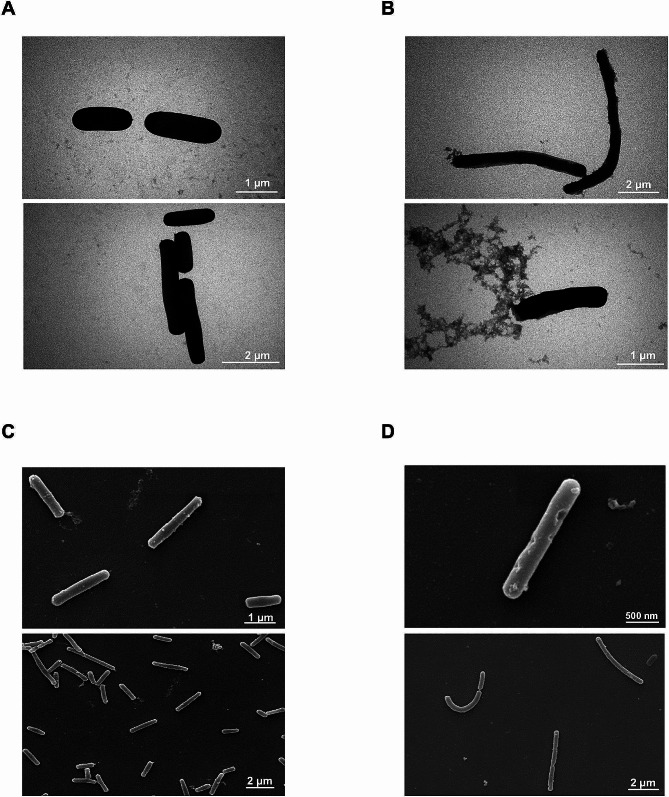
SEM and TEM observation of live and pasteurized *L. brevis* IBRC-M10790 bacteria. **(A)** TEM images of live bacteria. **(B)** TEM images of pasteurized bacteria. **(C)** SEM images of live bacteria. **(D)** SEM images of pasteurized bacteria

Appearance differences between live and pasteurized bacteria were investigated by TEM and SEM. As shown in Fig. 1, there were no noticeable morphological changes between live *L. brevis* and pasteurized bacteria, and both forms demonstrated intact structural integrity.

### Cytotoxicity evaluation of *L. Brevis* IBRC-M10790

**Fig. 2:**
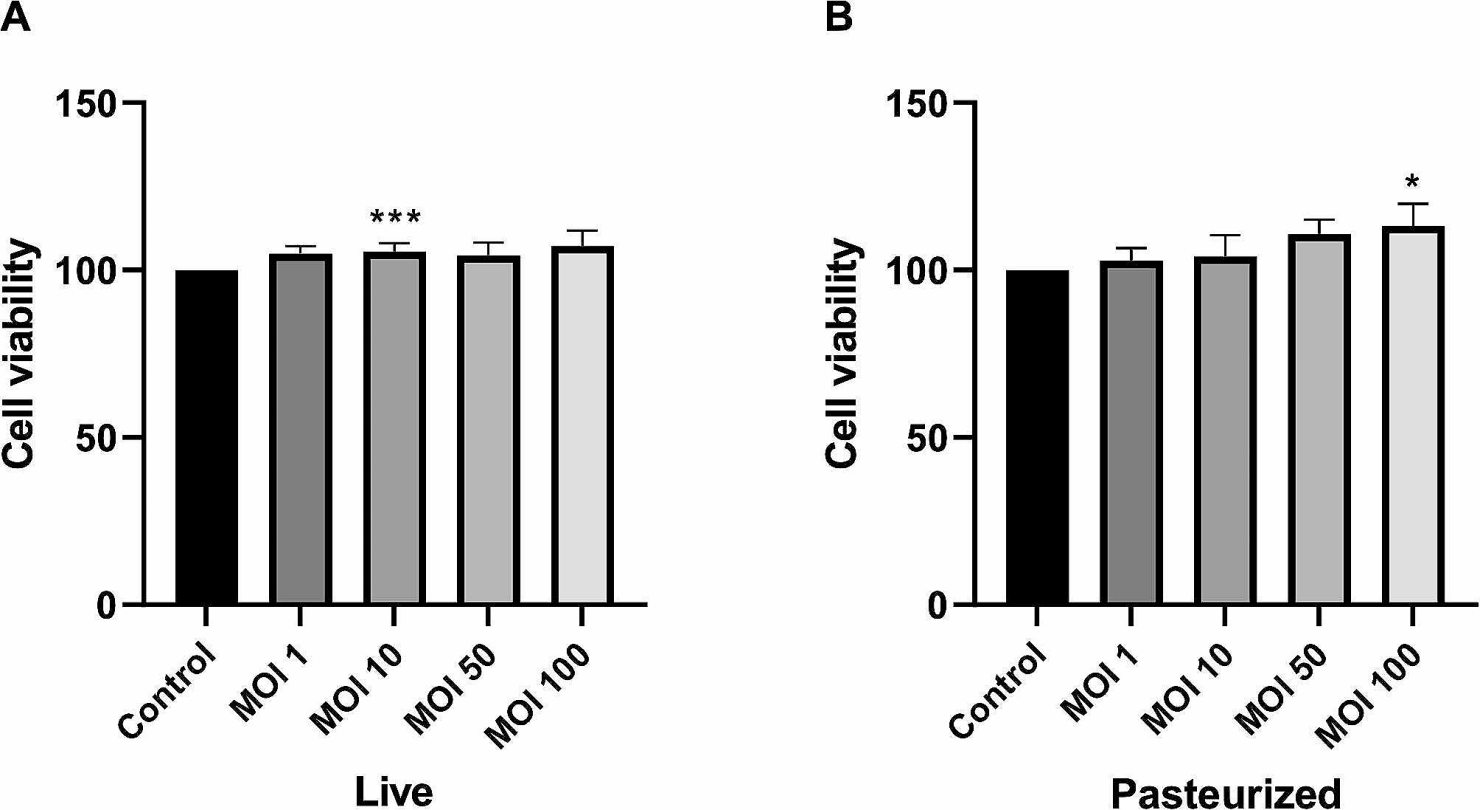
Viability percentage of Caco-2 cells treated with live and pasteurized *L. brevis* IBRC-M10790. The effect of **(A)** live and **(B)** pasteurized bacteria at different MOIs on cells was investigated. The numbers represent different MOIs; (1) control, (2) MOI 1, (3) MOI 10, (4) MOI 50, and (5) MOI 100. Control is untreated Caco-2 cells with 100% viability. The results are represented as Mean ± SEM. * and *** refer to the adjusted *P*-values < 0.05 and < 0.001

To evaluate cell viability, various MOIs of live and pasteurized *L. brevis* IBRC-M10790 were applied to Caco-2 cells. As shown in Fig. 2, the results indicated that at all MOIs, cell viability was not decreased and both live and pasteurized *L. brevis* showed no cytotoxic effects on Caco-2 cells. Among different MOIs (1, 10, 50, and 100) compared to the control, MOI 10 of live bacteria had a significant effect on the cell proliferation (*P*-value < 0.001) and was used for further cell experiments.

### Effects of *L. Brevis* IBRC-M10790 on the levels of cytokines

**Fig. 3:**
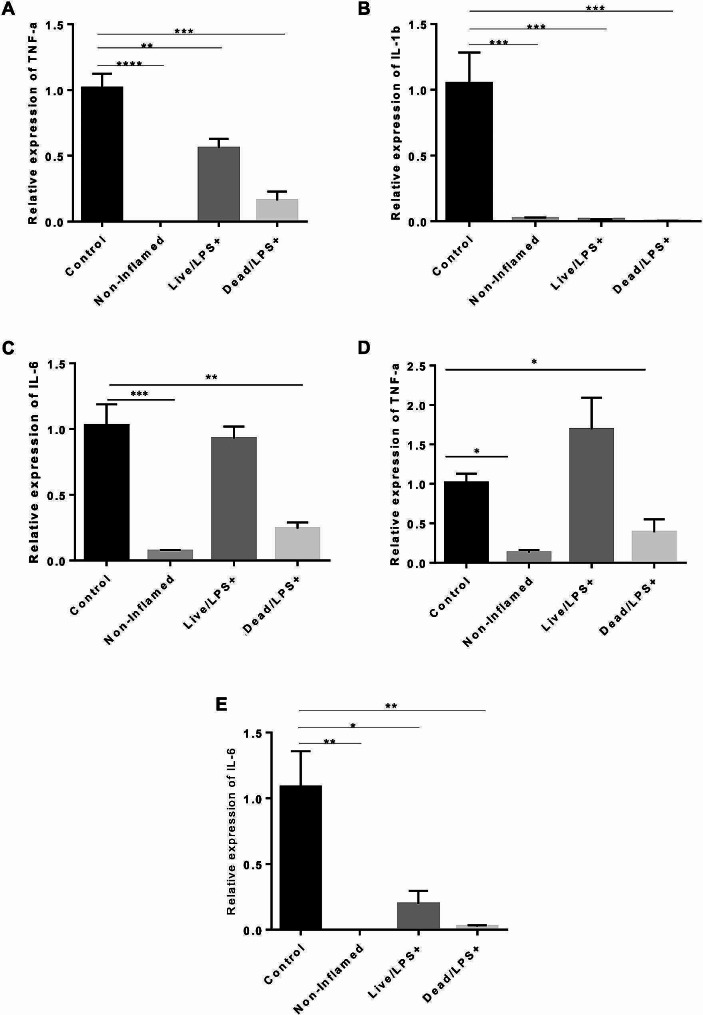
The effects of live and pasteurized*L. brevis * IBRC-M10790 on the mRNA levels of cytokines in the co-culture system treated with LPS. Relative mRNA expression levels of **(A) **TNF-α, **(B)** IL-1𝛽, and **(C)** IL-6 in Caco-2 cells, **(D) **TNF-α, and **(E)** IL-6 in RAW264.7 cells were measured by RT-qPCR. (Control) represents the culture conditions treated with LPS and not-treated with *L. brevis*, (Non-inflamed) represents the culture conditions without any treatment, (Live/LPS) represents the culture conditions treated with LPS and live *L. brevis*, (Dead/LPS) represents the culture conditions treated with LPS and pasteurized *L. brevis*. The results are represented as Mean ± SEM. *, **, *** and **** refer to adjusted *P*-values < 0.05, < 0.01, < 0.001, and < 0.0001

To determine the effect of live and pasteurized *L. brevis* IBRC-M10790 on the immunomodulatory profiles of RAW264.7 and Caco-2 cells, the gene expression of cytokines was investigated by RT-qPCR in the in vitro model of IBD. The results showed that LPS induced an inflammatory response in RAW264.7 cells, and increased the expression of TNF-𝛼 (*P*-value < 0.0001), IL-6 (*P*-value < 0.005), and IL-1 𝛽 (*P*-value < 0.005) in Caco-2 cells compared to non-inflammatory conditions. This indicated that inflammation was stimulated in the co-culture system, and the in vitro model similar to IBD was developed (Fig. 3).

**Fig. 4:**
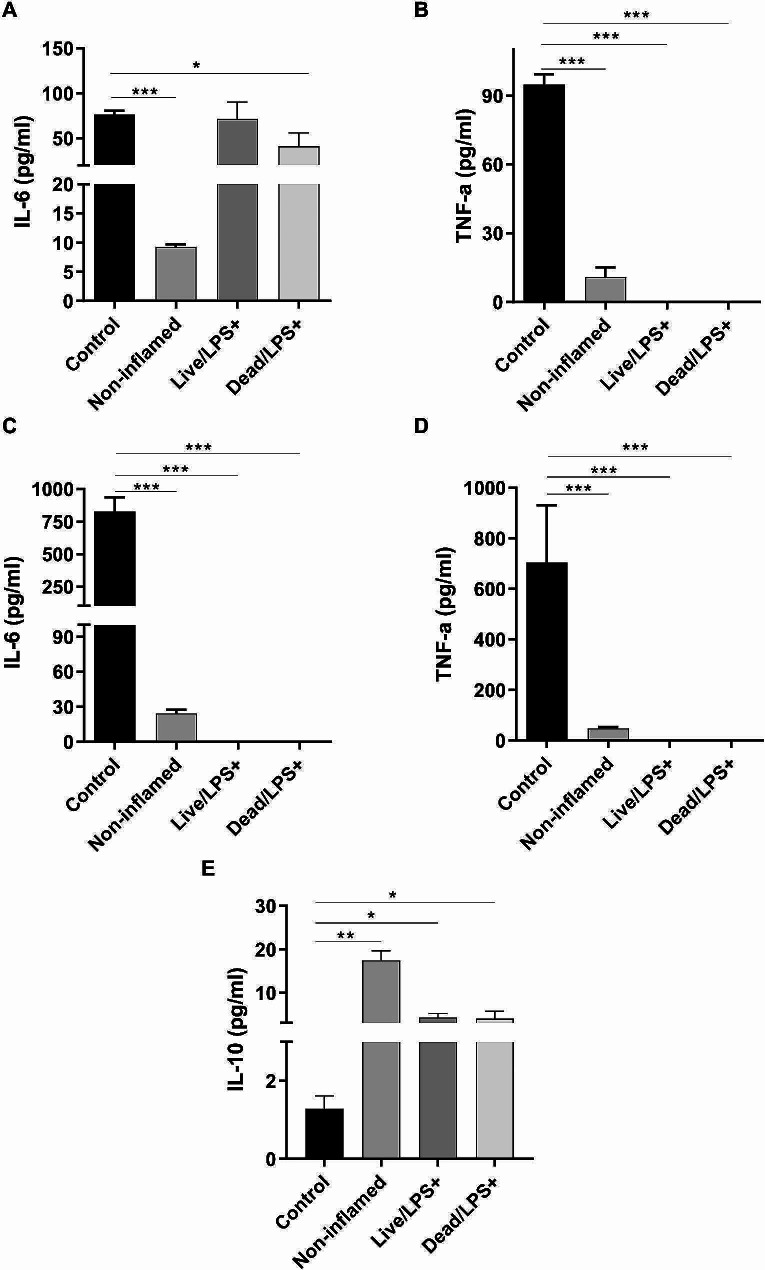
Effects of live and pasteurized*L. brevis*IBRC-M10790 on the level of cytokines in the co-culture system treated with LPS. The production of **(A)** IL-6 and** (B)** TNF-α in Caco-2 cells, **(C)** IL-6,** (D)** TNF-α, and **(E)** IL-10 were analyzed in RAW264.7 cells using ELISA. (Control) represents the culture conditions treated with LPS and not-treated with *L. brevis*, (Non-inflamed) represents the culture conditions without any treatment, (Live/LPS) represents the culture conditions treated with LPS and live *L. brevis*, (Dead/LPS) represents the culture conditions treated with LPS and pasteurized *L. brevis*. The results are represented as Mean ± SEM. *, **, and *** refer to the adjusted *P*-values < 0.05, < 0.01, and < 0.001

To examine the effect of live and pasteurized *L. brevis* IBRC-M10790 on the LPS-stimulated co-culture, the expression level of cytokines was measured. Our findings indicated that mRNA levels of proinflammatory cytokines including TNF-𝛼 (*P*-values < 0.01 and < 0.001) and IL-1𝛽 (*P*-values < 0.001 and < 0.001) significantly decreased in the epithelial cells (Fig. 3A-C) after treatment with live and pasteurized bacteria while the expression of IL-6 (*P*-values < 0.01) was substantially changed only in pasteurized *L. brevis*-treated epithelial cells. It was also observed that pasteurized *L. brevis* IBRC-M10790 caused higher reduction level in the expression of proinflammatory cytokines TNF-𝛼 (*P*-values < 0.05) and IL-6 (*P*-values < 0.01) compared to live bacteria in macrophages (Fig. 3D-E). To assess the effect of live and pasteurized bacteria on the cytokine production, their expression levels were also evaluated using ELISA. Firstly, ELISA results also corroborated that inflammatory conditions were significantly induced by LPS treatment in the co-culture system and IBD was modeled in vitro (Fig. 4). Furthermore, treatment with pasteurized *L. brevis* decreased the production of proinflammatory cytokines IL-6 and TNF-α in the intestinal epithelial cells (*P*-values < 0.05 and *P*-values < 0.001, respectively) (Fig. 4A-B) and macrophages (*P*-values < 0.001 and *P*-values < 0.001, respectively) (Fig. 4C-D), while increased the level of anti-inflammatory cytokine IL-10 compared to the control conditions (*P*-values < 0.05) (Fig. 4E). A similar pattern was also observed in cells treated with live bacteria (Fig. 4B-D), except that no significant change in IL-6 secretion was detected in epithelial cells (Fig. 4A).

### The effects of *L. Brevis* IBRC-M10790 on adherens and tight junctions

**Fig. 5:**
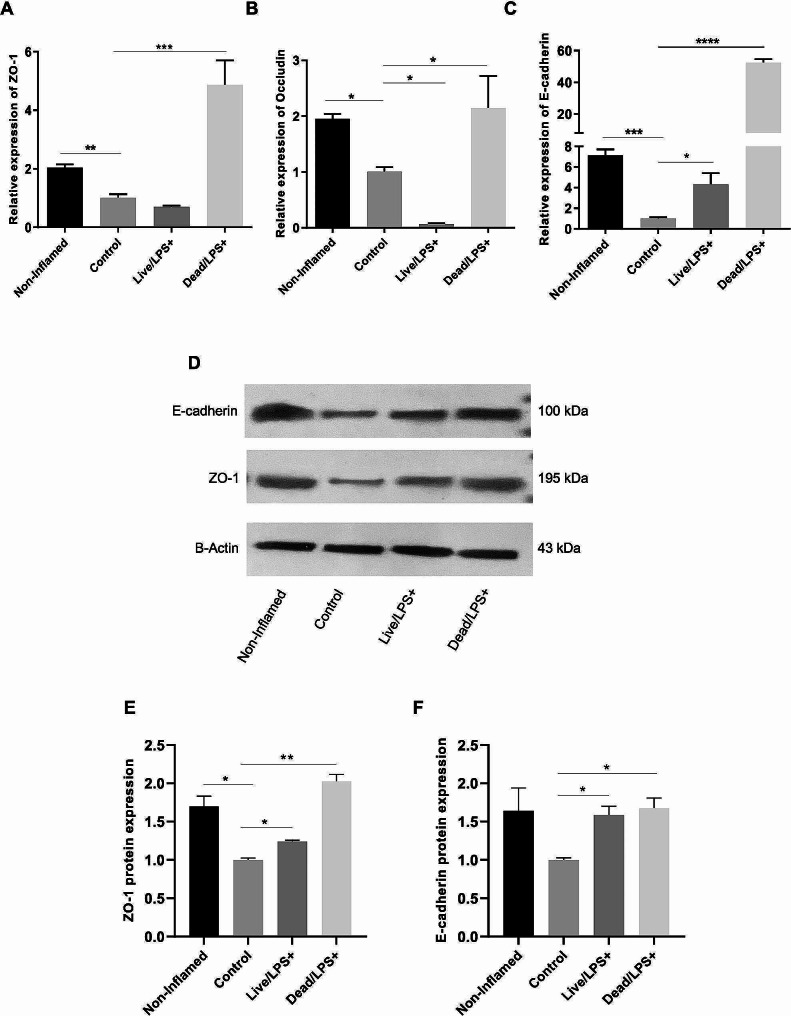
Effects of live and pasteurized*L. brevis* IBRC-M10790 on the expression levels of genes involved in intestinal barrier function. Genes expression changes of **(A)** ZO-1, **(B)** Occludin, and **(C) **E-cadherin transcripts after *L. brevis* IBRC-M10790 treatment. Protein expression changes of (**D-E**) ZO-1, and **(D and F)** E-cadherin s after *L. brevis* IBRC-M10790 treatment. (Control) represents the culture conditions treated with LPS and not-treated with *L. brevis*, (non-inflamed) represents the culture conditions without any treatment, (Live/LPS) represents the culture conditions treated with LPS and live *L. brevis*, (Dead/LPS) represents the culture conditions treated with LPS and pasteurized *L. brevis*. The results are represented as Mean ± SEM. *, **, *** and **** refer to the adjusted *P*-values < 0.05, < 0.01, < 0.001, and < 0.0001

To understand the mechanisms related to the protective effects of live and pasteurized *L. brevis* IBRC-M10790 on IBD, the expression of adherens and tight junctions (TJ) genes involved in intestinal barrier function was investigated in Caco-2 cell line. The results indicated that LPS treatment decreased the expression level of ZO-1 (*P*-values < 0.01), Occludin (*P*-values < 0.05), and E-cadherin (*P*-values < 0.001) similar to what is observed in IBD. Intriguingly, in the presence of pasteurized *L. brevis*, these three epithelial markers showed an increase compared to the control (*P*-values < 0.001, < 0.05, and < 0.0001, respectively), while live strain only upregulated E-cadherin (*P*-values < 0.0001). (Fig. 5A-C). In addition, the western blot analysis indicated that LPS treatment led to decreased expression levels of ZO-1 and E-cadherin proteins, while the presence of *L. brevis* could prevent the downregulation of adherens and tight junction proteins under the inflammation condition (Fig. 5D-F and Supplementary Fig. 1). However, our results also demonstrated that pasteurized form of *L. brevis* IBRC-M10790 could exert higher protective effect on epithelial barrier than live bacteria.

## Discussion

**Fig. 6:**
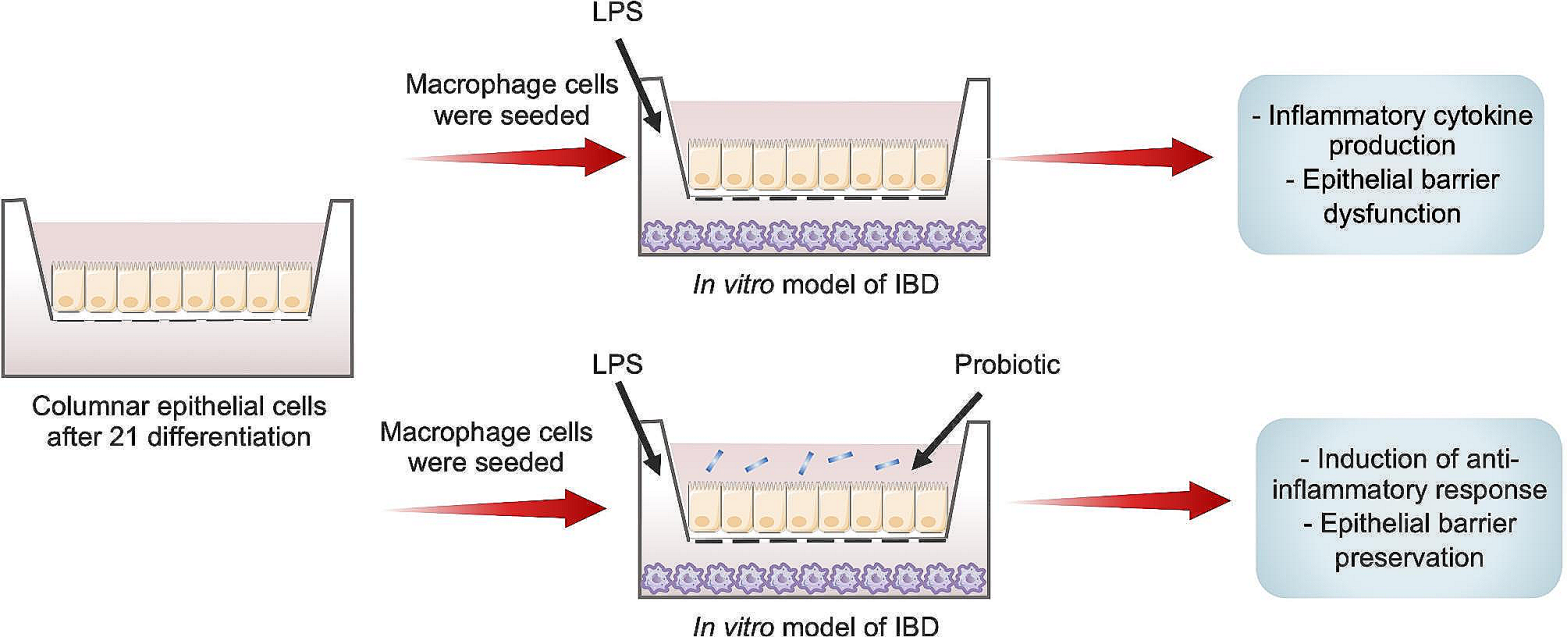
Probiotic effects of *L. brevis*IBRC-M10790 in IBD. Schematic representation of the in vitro modeling of IBD. Caco2 cells were cultured on the apical sides of Transwell inserts. After 21 days of differentiation, macrophages were cultured in the basolateral side and exposed to LPS. Treatment of *L. brevis* IBRC-M10790 exerted anti-inflammatory effect and led to preservation of epithelial barrier

Although genetic and environmental factors have been introduced as risk factors of IBD [39], today, it has been shown that dysbiosis, or imbalance in the composition of the gut microbiota is a key factor in the pathogenesis of IBD [40]. As no definitive treatment is currently available for this disease, it is important to identify new and safe strategies to improve gut microbiota structure and function, and modulate immune system responses to manage IBD [41]. Recently, the use of probiotics has been developed as a promising approach to mitigate IBD symptoms [42]. Therefore, it is necessary to identify and investigate different probiotic strains to obtain new therapeutic tools for the management of IBD [15]. *Lactobacillus* strains that are used as an indicator of a healthy gut, could be one of the important probiotics to improve gastrointestinal disorders such as IBD [13]. One of the most crucial characteristics of probiotic bacteria is their tolerance to acid and bile conditions because the survival of probiotics in the gastrointestinal tract depends to a large extent on their tolerance to acid and bile salts. Bile salts cause hydrolysis of fats and lipids in the bacterial cell membranes and can lead to their destruction [43, 44]. Based on the results, it was shown that *L. brevis* IBRC-M10790 has the potential for probiotic applications by tolerating acidic pH and bile salt conditions. In this study, live and pasteurized forms of *L. brevis* were used to evaluate their anti-inflammatory activity and also protective function on the integrity of intestinal barrier in a co-culture system of epithelial-like Caco-2 cells and LPS-treated RAW264.7 macrophages. This model is a cost-effective and high-performance laboratory system that, while simulating the gut-luminal interface, also provides the possibility of studying epithelial-immune interactions and recreates the key features of IBD such as gut inflammation (Fig. 6) [45]. Previously, in many studies, this type of co-culture system has been used to specify the anti-inflammatory role of various factors in IBD [46, 47]. In this study, Caco-2 cells were differentiated for 21 days to form a polarized monolayer with TJ and simulate the intestinal epithelial barrier. Also, RAW264.7 macrophages have been used as an in vitro model for research on inflammatory mediators. These cells are sensitive to LPS and are suitable for investigating the anti-inflammatory effects of probiotics [48, 49]. LPS stimulates RAW264.7 cells and increases the secretion of cytokines such as IL-1β, IL-6, and TNF-α, which induce inflammatory conditions [35]. Also, in this study, it was observed that LPS induced inflammatory conditions in the co-culture system by RAW264.7 cells, which affected the Caco-2 monolayer and increased inflammation (Fig. 6).

Another important characteristic of probiotics could be their capability to reduce proinflammatory cytokines secreted by the immune system [50]. It has been proven that high levels of proinflammatory mediators are secreted in patients with gut inflammation [51]. Inflammatory mediators are overproduced by macrophages in many clinical disorders, such as IBD. In patients with IBD, the immune system induces the secretion of proinflammatory cytokines IL-6, TNF-α, and IL-1β [52]. It has been shown that excessive production of these cytokines plays a key role in the pathogenesis of IBD. Accordingly, a reduction in the expression and secretion of these cytokines is recommended as a useful therapeutic strategy to manage IBD [26].

Many studies have shown that probiotics reduce the expression of proinflammatory cytokines in the in vitro and in vivo IBD models [53–55]. As shown in this study, the presence of LPS increased the proinflammatory cytokines TNFα, IL-1β, and IL-6 in control conditions, and *L. brevis* IBRC-M10790 could decrease proinflammatory cytokines in the inflamed cells at transcript and protein levels. This finding was consistent with previous results that reported the use of probiotic strains *L. brevis* Bmb6 and *L. brevis* K65 in a murine model with colitis reduced proinflammatory cytokines and preserved the structural integrity of the intestinal epithelium [13, 24].

In this study, comparing the effects of live and pasteurized *L. brevis* IBRC-M10790 on intestinal epithelial cells indicated that despite the significant effect of both forms in reducing proinflammatory cytokines, pasteurized *L. brevis* had a greater effect on reducing IL-1β, IL- 6, and TNF-α. These results were in agreement with the study of Liu et al., also reported the beneficial effects of both live and heat-killed *L. brevis* K65 in macrophage cells by reducing the expression of TNF-α, nitric oxide, and prostaglandin E2 [24]. It should be noted that, unlike live probiotics, pasteurized probiotics do not need to be colonized in the intestinal lumen to survive and maintain their activities, and this is an advantage of using pasteurized forms because it is possible that live probiotics cannot be properly colonized in the intestinal tract of a person with IBD. Heat-killed bacteria could also help to maintain intestinal homeostasis and treat intestinal inflammation in a dextran sodium sulfate (DSS) colitis model [25].

Another characteristic of probiotics is their ability to induce the secretion of anti-inflammatory cytokine IL-10, which leads to the reduction of intestinal inflammation [53]. This study indicated that the presence of *L. brevis* IBRC-M10790 in the co-culture system stimulated the production of IL-10 compared to the control conditions, which can help alleviating the intestinal inflammation.

The intestinal barrier, which consists of adherens and TJ proteins, protects the homeostasis of the gut microbiota and the immune system. These junctions connect intestinal epithelial cells and adjust permeability. Occludin, ZO-1, and E-cadherin proteins are among the important markers whose sufficient expression protect proper intestinal permeability, and downregulation leads to IBD progression [35]. In the present study, it was shown that the expression level of Occludin, ZO-1, and E-cadherin decreased upon LPS induction, while in the presence of pasteurized *L. brevis*, these markers were significantly upregulated. Previous studies have also shown that the use of probiotics increases the expression of genes involved in adherens junctions and TJ [56].Potential probiotics in IBD can impose barrier protection effects through different mechanisms. Liu et al. reported that specific strains of *Lactobacillus* could maintain intestinal barrier function via cytoprotective heat shock proteins (HSPs) induction, and may also protect the host against the effects of pathogenic bacteria through the regulation of TJ proteins and inhibition of epithelial barrier disruption [57]. Anderson et al. showed that probiotic *Lactobacillus plantarum* MB452 can Increase the mucosal integrity through enhancing the expression levels of genes engaged in adherens and TJ.

Our findings highlighted that *L. brevis* IBRC-M10790 has a high potential as a probiotic that could improve IBD through its immunomodulatory activity, and also preserve the expression of adherens and TJ markers that regulate the integrity of the intestinal epithelial barrier (Fig. 6).

## Conclusion

In conclusion, we demonstrated that *L. brevis* IBRC-M10790 caused a significant decrease in the amount of pro-inflammatory cytokines, including IL-6, IL-1β, and TNF-α, and it also increased the anti-inflammatory cytokine IL-10 in in vitro co-culture model of IBD. In addition, *L. brevis* increased the expression of TJ proteins (ZO-1, Occludin, and E-cadherin), which can maintain the epithelial characteristics and mucosal integrity of the epithelial cell monolayers. Furthermore, we showed that the use of pasteurized *L. brevis* is preferable to live *L. brevis*, particularly in preventing the suppression of epithelial markers in the inflammatory conditions. Overall, the findings of this study suggest that *L. brevis* IBRC-M10790 as a probiotic bacterium with high potential can be a therapeutic agent to manage IBD, although further in vivo studies are needed to apply in clinical settings. Considering the positive results of the probiotic effect of *L. brevis* bacteria on the co-culture model of Caco-2 and RAW264.7 cells, it is necessary to conduct more in-depth research to confirm the findings of this study. Despite the advantages of in vitro models, their limitations make them unable to fully reflect the natural environment of the intestine, such as the complexity of the human gut microbiome and immune system. Therefore, it is needed to confirm the probiotic effect of *L. brevis* IBRC-M10790 on more complex systems that better recapitulate the human gut pathophysiology, such as colitis-induced animal models.

### Electronic supplementary material

Below is the link to the electronic supplementary material.

Supplementary Material 1

## Data Availability

The generated data during this study are included in the article. Further data are available from the corresponding author at a reasonable request.
