# Supplementary material for: Pasteurized form of a potential probiotic lactobacillus brevis IBRC-M10790 exerts anti-inflammatory effects on inflammatory bowel disease in vitro
Source: BMC Complement Med Ther. 2024 Jul 10;24:258. doi: 10.1186/s12906-024-04576-1 (PMC11234635; doi:10.1186/s12906-024-04576-1)
Supplement: Supplementary file 1 — Supplementary Material 1 [file 12906_2024_4576_MOESM1_ESM.pdf]

Pasteurized form of a potential probiotic *lactobacillus brevis* IBRC-M10790 exerts anti-inflammatory effects on inflammatory bowel disease *in vitro*

Ardeshir Ebrahiminejad[1], Abbas Akhavan Sepahi[1], Abbas Yadegar[2*], Anna Meyfour[3*]

1. Department of Microbiology, Faculty of Biological Sciences, Islamic Azad University, North Tehran Branch, Tehran, Iran
2. Foodborne and Waterborne Diseases Research Center, Research Institute for Gastroenterology and Liver Diseases, Shahid Beheshti University of Medical Sciences, Tehran, Iran
3. Basic and Molecular Epidemiology of Gastrointestinal Disorders Research Center, Research Institute for Gastroenterology and Liver Diseases, Shahid Beheshti University of Medical Sciences, Tehran, Iran

*Corresponding authors:

Anna Meyfour, Research Institute for Gastroenterology and Liver Diseases, Shahid Beheshti University of Medical Sciences, Arabi Ave., Daneshjoo Blvd., Velenjak, Tehran, Iran, Postal Code: 1985717413, Tel: +98 21 22432521 (a.meyfour@sbmu.ac.ir).

Abbas Yadegar, Foodborne and Waterborne Diseases Research Center, Research Institute for Gastroenterology and Liver Diseases, Shahid Beheshti University of Medical Sciences, Shahid Arabi Ave., Yemen St, Velenjak, Tehran, Iran, Postal Code: 1985717413, Tel: +98 21 22432521 (a.yadegar@sbmu.ac.ir).

A. Anti- E-cadherin

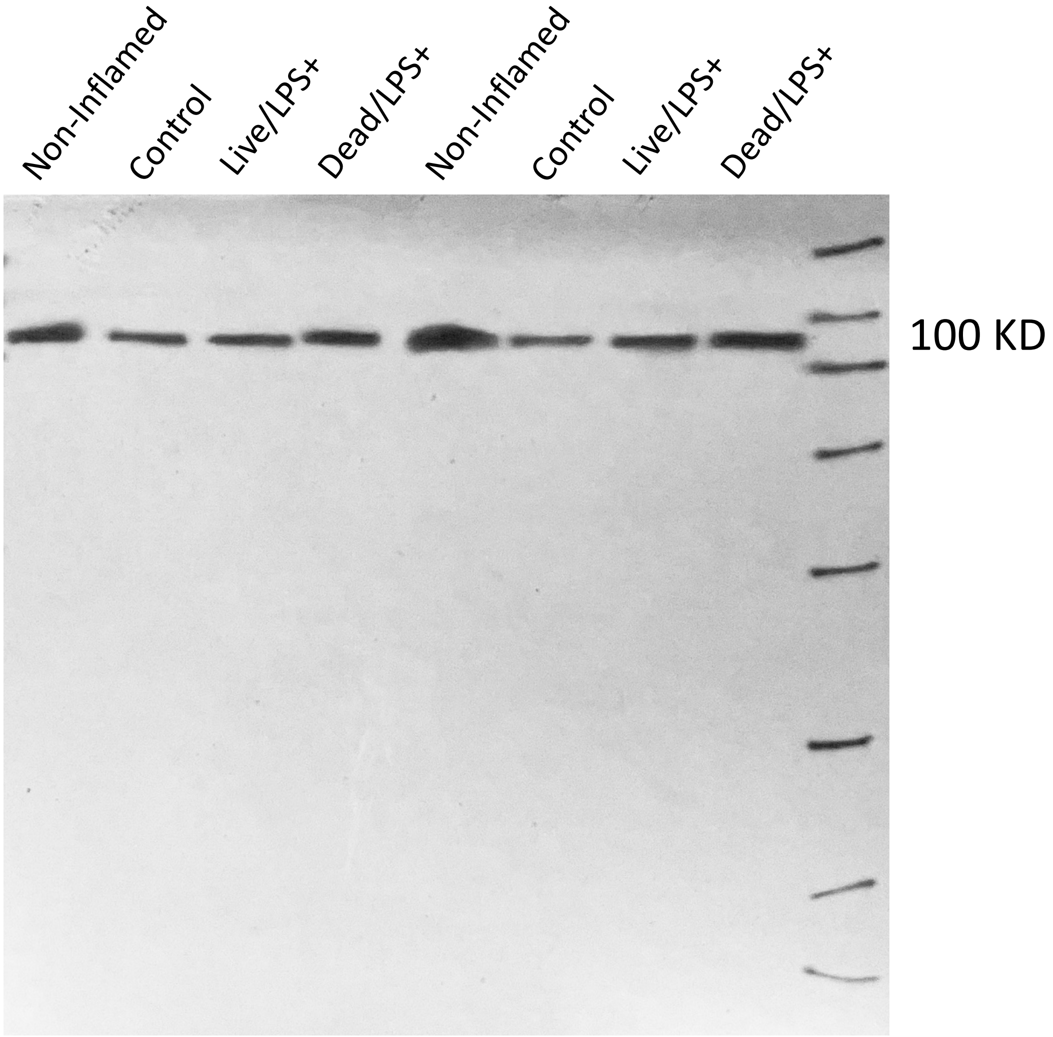

B. Anti- ZO-1

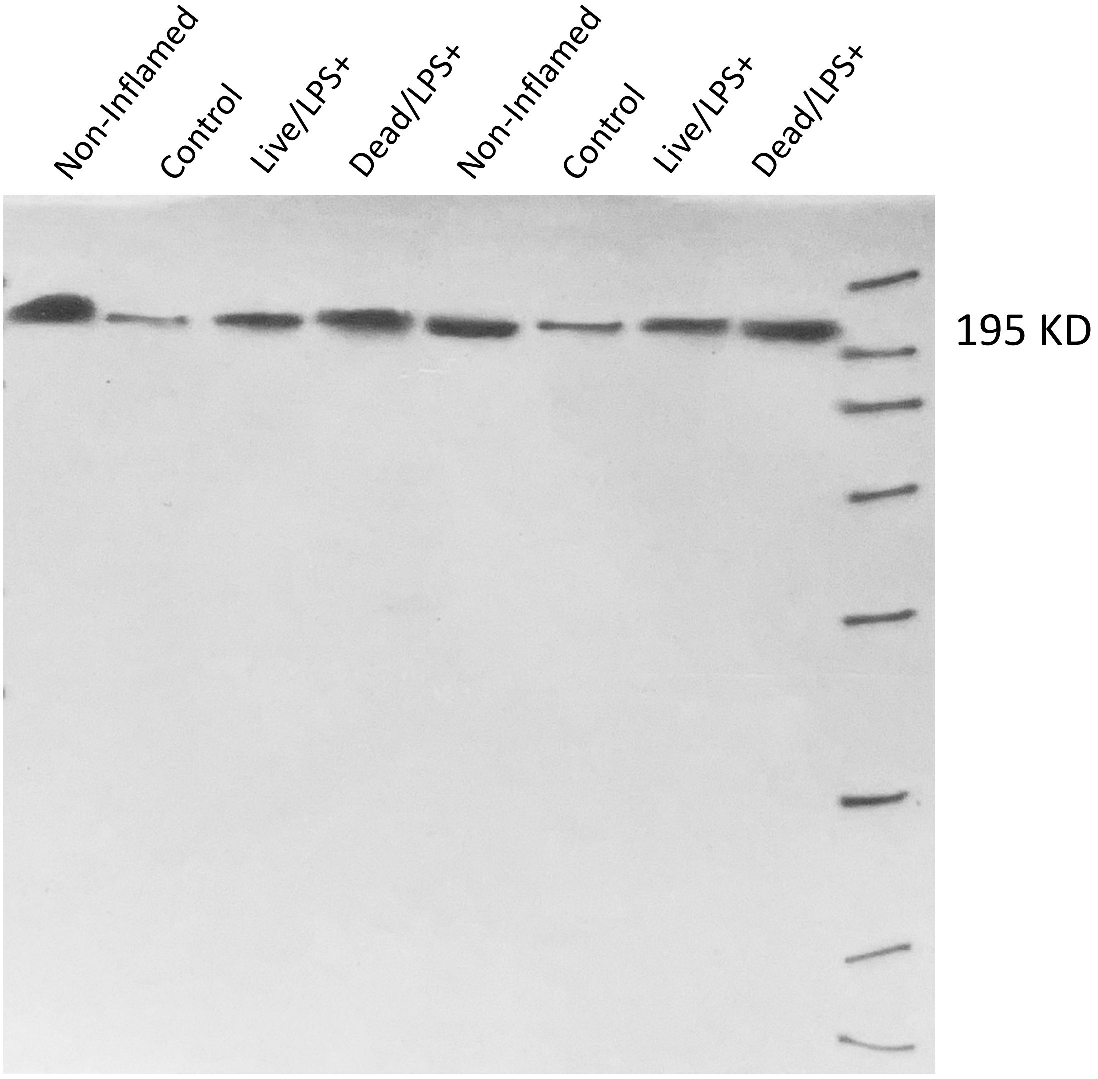
Non-Inflamed
Control
Live/LPS+
Dead/LPS+
Non-Inflamed
Control
Live/LPS+
Dead/LPS+
195 KD

C. Anti- β-Actin

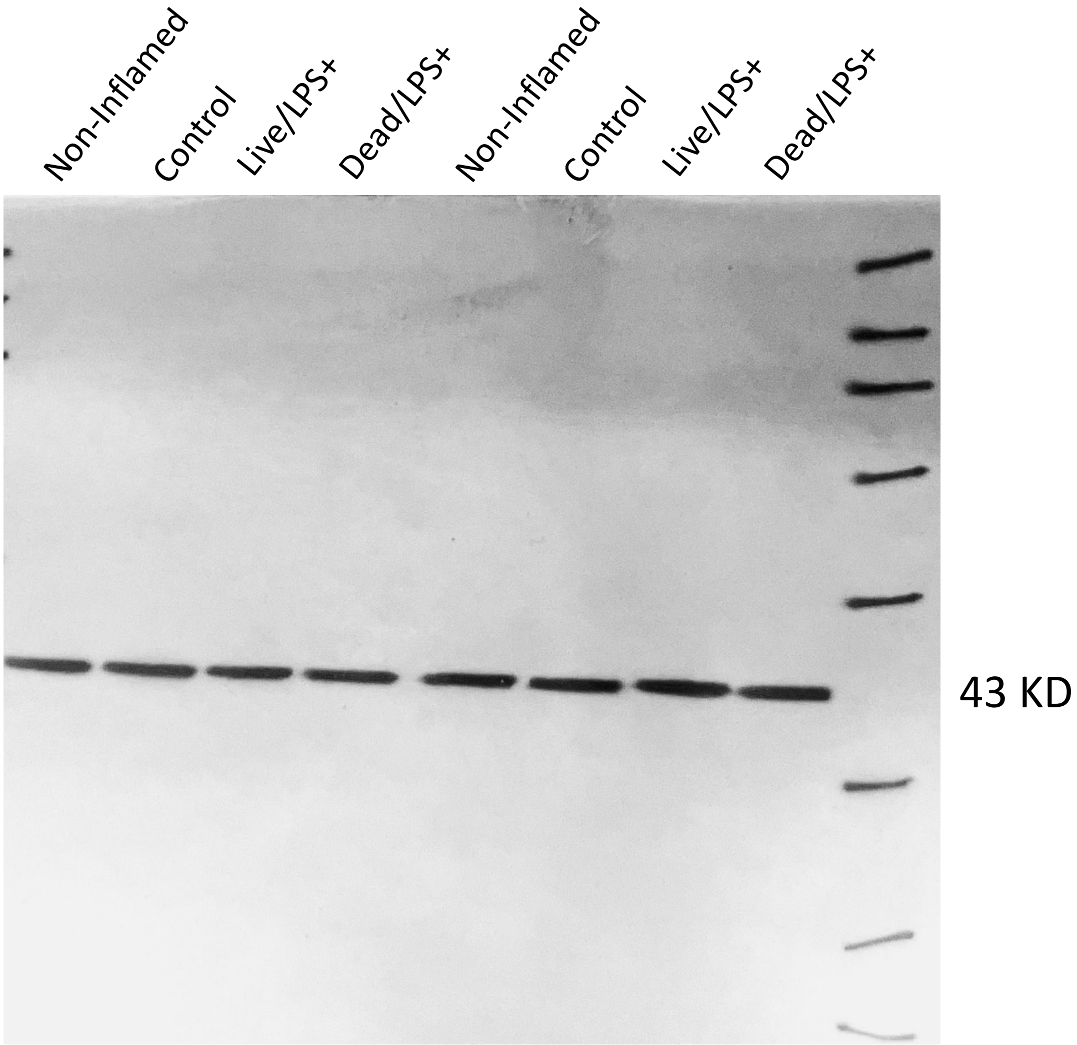

Supplementary Figure 1. Western blotting analysis to detect (A) E-cadherin, (B) ZO-1, and (C) β-Actin. Non-Inflamed represents the culture conditions without any treatment, Control represents the culture conditions treated with LPS and not-treated with *L. brevis*, Live/LPS$^+$ represents the culture conditions treated with LPS and live *L. brevis*, and Dead/LPS$^+$ represents the culture conditions treated with LPS and pasteurized *L. brevis*.
